# 1192. Correlation Between SARS CoV 2 Viral Load and Clinical Evolution of Patients Under 15 Years of Age with COVID 19 in a General Hospital in the Province of Buenos Aires, Argentina

**DOI:** 10.1093/ofid/ofab466.1384

**Published:** 2021-12-04

**Authors:** Martin Brizuela, Sandra Goñi, Georgina Cardama, Leandro Sommese, Hernan Farina

**Affiliations:** 1 Hospital Zonal General de Agudos “Dr Isidoro G Iriarte”, Buenos Aires, Ciudad Autonoma de Buenos Aires, Argentina; 2 Universidad Nacional de Quilmes, Quilmes, Buenos Aires, Argentina

## Abstract

**Background:**

SARS CoV2 infection produces clinical manifestations of different severity. The pediatric population represents less than 10% of cases, with a mortality of less than 1%. The severity of the condition and mortality are mainly associated with comorbidities. There is controversy about the correlation between the viral load of SARS CoV2 in respiratory samples and the evolution and severity of the clinical picture. The CT (cycle threshold) in the detection of the SARS CoV 2 genome in respiratory samples can be used as an indirect indicator of the viral load in the analyzed samples.

**Goals:**

to determine the correlation between the SARS CoV 2 CT values in the detection of the viral genome with the severity of the clinical picture. Describe the clinical, epidemiological, and laboratory characteristics of patients with PCR-confirmed SARS CoV2 infection in respiratory samples.

**Methods:**

A retrospective, observational and analytical study that included patients under 15 years of age with confirmed SARS CoV2 infection by PCR of respiratory samples treated at the Hospital Isidoro Iriarte in the city of Quilmes between March 1 2020 and April 30, 2021.

**Results:**

485 patients (n) were included. The distribution by severity of the clinical picture was mild (84%, n = 408), moderate (12%, n = 59) and severe (4%, n = 18). Comorbidities were more frequent among patients with moderate and severe symptoms. Viral load was associated with severity of clinical manifestations. Patients with moderate and severe COVID19 required hospital admission more frequently for a longer time, the use of supplemental oxygen and antibiotics were more frequent in patients with moderate and severe symptoms. Symptoms of lower respiratory tract infection such as cough and respiratory distress were more frequent in patients with moderate and severe symptoms. No patient required admission to the ICU or mechanical ventilation. No patient died.

**Conclusion:**

In this study, patients with moderate and severe COVID19 infection had a higher viral load in respiratory samples, a higher frequency of comorbidities, a higher frequency of hospitalization and a longer hospital stay. Lower respiratory symptoms were associated with moderate and severe symptoms, while odynophagia, vomiting, and diarrhea were associated with mild clinical symptoms.

**Disclosures:**

**All Authors**: No reported disclosures

